# Energy-Dense Diets and Mineral Metabolism in the Context of Chronic Kidney Disease–Metabolic Bone Disease (CKD-MBD)

**DOI:** 10.3390/nu10121840

**Published:** 2018-12-01

**Authors:** Mariano Rodriguez, Escolastico Aguilera-Tejero

**Affiliations:** 1 Maimonides Biomedical Research Institute of Cordoba (IMIBIC), Reina Sofia University Hospital, University of Cordoba, 14004 Cordoba, Spain; marianorodriguezportillo@gmail.com; 2 Department Medicina y Cirugia Animal, University of Cordoba, 14071 Cordoba, Spain

**Keywords:** diet, calories, fat, CKD-MBD

## Abstract

The aim of this paper is to review current knowledge about the interactions of energy-dense diets and mineral metabolism in the context of chronic kidney disease–metabolic bone disease (CKD-MBD). Energy dense-diets promote obesity and type II diabetes, two well-known causes of CKD. Conversely, these diets may help to prevent weight loss, which is associated with increased mortality in advanced CKD patients. Recent evidence indicates that, in addition to its nephrotoxic potential, energy-dense food promotes changes in mineral metabolism that are clearly detrimental in the context of CKD-MBD, such as phosphorus (P) retention, increased concentrations of fibroblast growth factor 23, decreased levels of renal klotho, and reduction in circulating concentrations of calcitriol. Moreover, in uremic animals, a high fat diet induces oxidative stress that potentiates high P-induced vascular calcification, and these extraskeletal calcifications can be ameliorated by oral supplementation of vitamin E. In conclusion, although energy-dense foods may have a role in preventing undernutrition and weight loss in a small section of the CKD population, in general, they should be discouraged in patients with renal disease, due to their impact on P load and oxidative stress.

## 1. Introduction

Metabolic syndrome (MS) is characterized by obesity and its associated complications, like insulin resistance, dyslipidemia, and hypertension [1]. Obesity and, in general, MS, have a multifactorial origin that includes: genetic predisposition, sedentary life style, and excess caloric intake [2]. Obesity, by itself or in the context of MS, is known to be harmful to the kidneys [3]. Therefore, eating energy-dense foods, which may predispose to obesity and MS, would seem inadequate for patients with chronic kidney disease (CKD). 

However, in patients with end-stage renal disease in renal replacement therapy, a high caloric intake may be recommended to prevent negative nitrogen balance and to reduce urea generation [4]. In addition, weight loss and malnutrition can be a serious problem in CKD patients, particularly in aged individuals and those with co-morbidities. Weight loss starts early in the course of renal disease, and is associated with the progression of CKD and mortality [5]. Defective hypothalamic appetite regulation promotes anorexia in uremic patients [6]. Moreover, poor appetite is often aggravated by dietary restrictions that are primarily aimed at reducing phosphorus (P) and potassium intake, but that negatively affect food palatability. Therefore, energy-dense foods may be indicated in renal patients, not only because of their high caloric content, but also because of their tastiness. Strategies using energy-dense meals or supplementation with snacks have been found to be useful to prevent undernutrition in hospitalized geriatric patients [7]. Thus, high caloric intake may be helpful in an attempt to neutralize the malnutrition of patients in different CKD stages.

**Figure 1:**
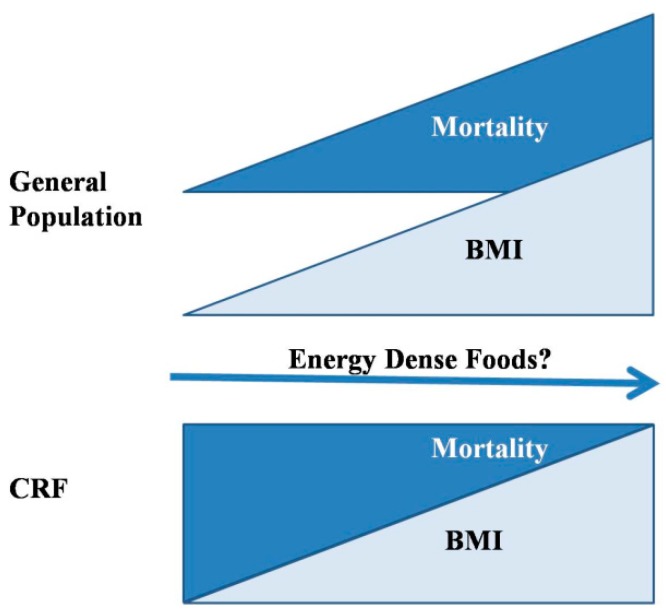
The obesity paradox. While in the general population, increases in body weight and body mass index (BMI) are directly correlated with mortality, in patients with chronic renal failure (CRF), an inverse correlation is observed. Since eating energy-dense foods is associated with increases in BMI, these diets are likely to result in increased mortality in the general population, but what is their effect in the CRF population?

It is interesting to note that scientific evidence not only indicates that weight loss is prejudicial, but also shows that overweightness may be beneficial for CKD patients. This is known as the obesity paradox, and is based on the fact that, although obesity is detrimental for the kidney, mortality in CKD patients is inversely proportional to body weight and body mass index (BMI) [8]. While in the general population, increases in BMI from 25 to 40 are accompanied by a steady increase in mortality, the opposite occurs in patients with CKD. In the population of advanced predialysis (estimated glomerular filtration rate (eGFR) < 30 mL/min/1.73 m^2^), individuals within the overweight and obese ranges of BMI have greater survival rates than patients with lower BMI [9]. Thus, overweight CKD patients seem to be paradoxically protected against mortality (Figure 1). 

Renal disease is accompanied by a series of disorders in mineral metabolism. Elevations in fibroblast growth factor 23 (FGF23) and increased secretion of parathyroid hormone (PTH) help to prevent the accumulation of phosphate and hypocalcemia. The high FGF23 concentrations reduce calcitriol production, and therefore, circulating calcitriol is also decreased [10]. When deterioration of renal function progresses, the kidneys are not able to handle a normal P load, and hyperphosphatemia ensues. Elevated concentrations of extracellular P are a major factor in the development of vascular calcification (VC) [11]. In addition, changes in mineral metabolism are often accompanied by bone disease. All of these CKD-associated derangements are collectively known as metabolic bone disease (MBD) [12]. Emerging evidence indicates that excessive caloric intake and obesity are also likely to promote bone disorders [13,14], although their influence on CKD-MDB is unclear [15]. 

The aim of this paper is to review current knowledge about the interactions of energy-dense diets and disturbances of mineral metabolism associated with CKD-MBD, with an emphasis on VC.

## 2. Energy-Dense Diets and Mineral Metabolism

There is a growing body of evidence linking obesity and energy-dense diets to mineral metabolism, and these associations are of great interest in the context of CKD-MBD.

### 2.1. Energy-Dense Diets and Calcium

The influence of energy density of the diet on calcium (Ca) status will be greatly dependent on the Ca content of the diet. Although this can be very variable, in general, energy-dense food tends to contain little Ca. Independent of the diet’s Ca concentration, high fat intake has been shown to consistently reduce the intestinal absorption of Ca, both in rodents [16] and humans [17]. The reason for the reduced Ca absorption is related to the interaction between Ca and lipids in the intestinal lumen, leading to the formation of Ca soaps [16]. Thus, eating energy-dense diets may cause a negative Ca balance, and eventually aggravate hypocalcemia in patients with CKD-MBD.

### 2.2. Energy-Dense Diets and Phosphorus

**Figure 2:**
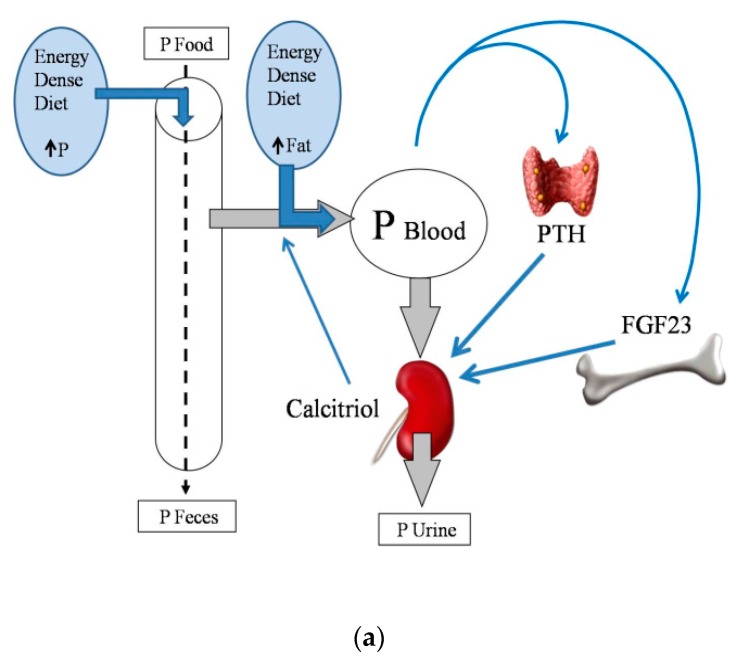

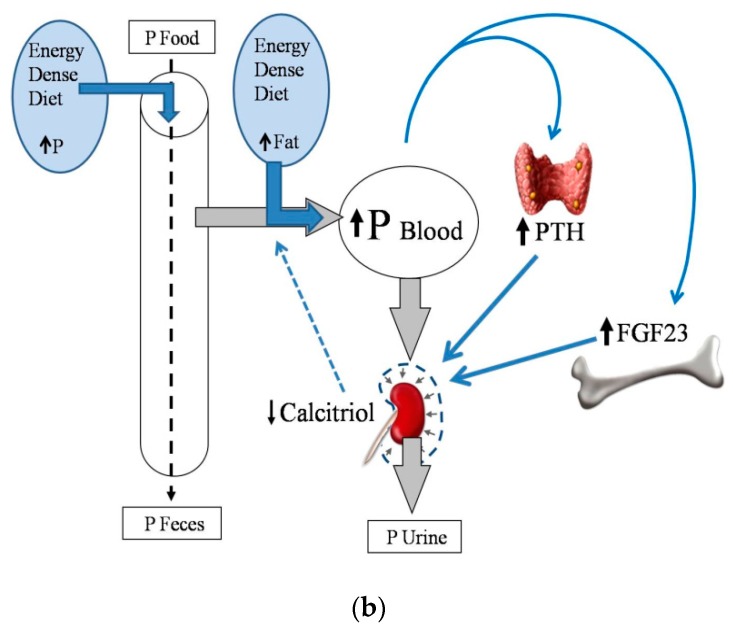
(**a**) Phosphate (P) balance in normal renal function; (**b**) Phosphate (P) balance in chronic renal failure. Fibroblast growth factor 23: FGF23; Parathyroid hormone: PTH.

Energy-dense diets are usually rich in P, which is widely used as a food additive. It is important to note that the inorganic P that is added to processed foods is more readily absorbed than the organic P that is naturally contained in foodstuffs [18]. Information on the actual P content of food is scant, and fast food is likely to represent a hidden source of dietary P intake [19]. Moreover, a high fat content in the diet has been reported to increase P digestibility [20], further aggravating P load. The influence of dietary fat on intestinal P absorption also seems to be secondary to the formation of Ca soaps in the intestinal lumen. Trapping of Ca in Ca soaps decreases the generation of insoluble Ca–P complexes, thus allowing more P ions to be free and able to be absorbed [20] (Figure 2a). 

According to the latest KDIGO (Kidney Disease Improving Global Outcomes) guidelines [12], CKD patients should have a normal serum P, and therefore, this should be maintained by a P-restricted diet, but the use of phosphate binders is not advised until serum P is elevated. Patients with MS and CKD often eat energy-dense diets; thus, it is important to know how obesity and a high fat diet may affect the handling of P when renal function is impaired. This has not been approached in clinical trials, but data obtained in experimental animals indicate that energy-dense diets would contribute to P retention in individuals with reduced renal function. A recent study has described that feeding a high fat diet for a short period of time (1 month) does not influence P balance in rats with intact renal function on a diet with normal P, but it leads to P retention when either renal function is decreased, or when the P content of the diet is increased [21].

Phosphorus overload is particularly deleterious in CKD-MBD patients, for its consequences on soft tissue mineralization and on the progression of renal disease. Therefore, in general, and with the exception of malnourished individuals, energy-dense diets should be considered as inadequate for CKD-MBD patients, due to their impact in P metabolism (Figure 2b).

### 2.3. Energy-Dense Diets and Magnesium

The inter-relationship between magnesium (Mg) and energy metabolism has been extensively studied. Obesity and MS are associated with Mg deficiency, and Mg replacement is known to improve the metabolic profile, both in the general population [22], and in patients with CKD [23]. Although processed food is typically low in Mg, a high fat intake does not influence Mg status in rats [24]. Magnesium may influence both carbohydrate and lipid metabolism, but in a different manner. Regarding carbohydrates, an inverse relationship between glycemic control and Mg levels has been described [22]. Conversely, low magnesium intake seems to protect against the deleterious consequences of high fat diets by reducing body weight [25] and adiposity [26] in experimental studies in rodents. Moreover, the reduced adiposity indirectly benefits glycemic control in these animals [26]. In the context of CKD-MBD, patients tend to have low plasma Mg, and hypomagnesemia has been correlated with increased mortality [27]. Moreover, Mg supplementation has been found to be effective for the prevention of uremic VC [28]. Thus, although reducing dietary Mg may have some favorable effects on animals eating high fat diets, adequate Mg intake is needed in CKD-MBD patients.

### 2.4. Energy-Dense Diets and PTH

To our knowledge, the direct effects of high calorie or high fat intake on the parathyroid glands have not been reported. However, the reduced Ca load and increased P load in response to feeding energy-dense foods could impact on the parathyroid glands, leading to the increased secretion of PTH.

In addition, energy-dense diets may have an indirect effect on PTH, mediated by hyperlipidemia and hyperleptinemia. The anabolic effects of PTH on bone are impaired in hyperlipidemic mice [29]. Also, increased plasma leptin, which is a consistent finding in obese individuals, has been shown to increase PTH secretion in rats [30].

In conclusion, all of the reported indirect effects of energy-dense diets on PTH point towards an increase in PTH, which would be counterproductive in CKD patients with secondary hyperparathyroidism.

### 2.5. Energy-Dense Diets and FGF23/Klotho

Fibroblast growth factor 23 has been reported to be elevated in obese people [31], and increased energy intake has been identified as a potential predictor of plasma FGF23 concentrations [32]. Recent studies in rats have demonstrated a direct relationship between the ingestion of a high calorie/high fat diet, and increases in the plasma concentrations of FGF23 [21,33]. Feeding high fat diets was consistently associated with increases in plasma FGF23 concentrations, both in rats that did not increase body weight [21], and in rats that increased body weight [33]. Thus, in these studies, the effect of a high fat diet on FGF23 seems to be independent of obesity. 

**Figure 3:**
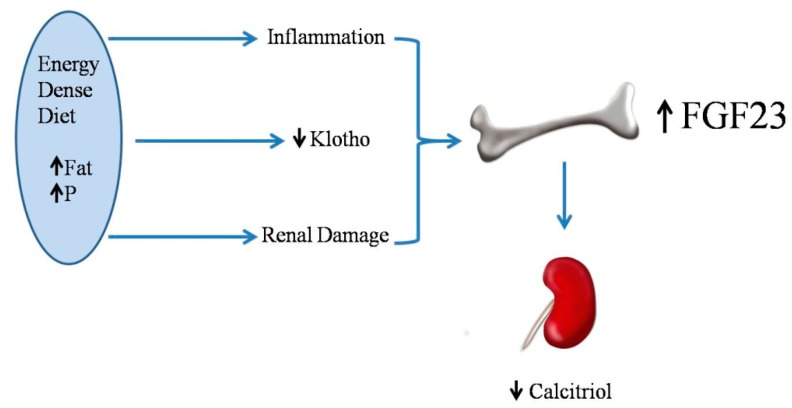
Energy-dense diets increase fibroblast growth factor 23 (FGF23) synthesis and secretion which in turn decreases calcitriol production. Phosphate: P.

The mechanism for increased FGF23 after feeding energy-dense diets is likely related to a decrease in renal klotho. Renal klotho has been shown to decrease in response to high fat diets in Wistar rats [21] and in apolipoprotein E (APoE) knockout mice [34]. When renal klotho is decreased, tubular resistance to FGF23 ensues, and more FGF23 is needed to maintain phosphaturia, consequently resulting in an increase in circulating levels of FGF23 [35]. In addition, high fat feeding and obesity may elicit systemic inflammation [36] and renal injury [37] which could also influence FGF23. Two recent studies have reported an association between increases in tumor necrosis factor alpha (TNFα) and FGF23 in rodents that are fed high fat diets [33,38] (Figure 3).

Adipokines may also influence the increase in FGF23 after feeding high fat diets. One factor that might be relevant is the increase in plasma leptin concentrations observed in obese individuals. Leptin, which is increased in rats that are fed high levels of fat, even before the development of obesity, has been shown to stimulate FGF23 secretion by osteocytes [39]. The effect of leptin is mediated by the up-regulation of the stimulatory action of calcitriol on skeletal synthesis of FGF23 [40]. Moreover, adiponectin signaling has been reported to reduce FGF23 secretion [41]. Since high fat intake is usually accompanied by reductions in plasma adiponectin [33] this may be another factor to explain the increase in FGF23 after eating energy-dense diets.

Elevated circulating levels of FGF23 promote progression of kidney disease and aggravate cardiovascular complications in patients with CKD [42,43]. Thus, the increase in FGF23 after feeding energy-dense diets is clearly deleterious in the context of CKD-MBD.

### 2.6. Energy-Dense Diets and Vitamin D

Low vitamin D levels have been reported in obese humans [44] and in obese rodents [45]. Moreover, feeding energy-dense diets results in decreased plasma calcitriol concentrations, even in animals that do not experience an increase in body weight [21]. When comparing rats that are fed high and normal fat diets with identical vitamin D contents, plasma calcitriol was significantly lower in rats that were fed high fat [21,33]. The influence of dietary fat on the intestinal absorption of vitamin D is somewhat controversial, but diets with high fat contents have been reported to increase vitamin D absorption by the intestine [46]; thus, the decreased absorption of vitamin D is unlikely in rats that are fed high fat. Moreover, plasma calcidiol concentrations were not decreased in rats that were fed high fat diets [21]. Since calcidiol is the metabolite that best reflects the nutritional status for vitamin D [47], the origin of the decreased plasma calcitriol cannot be attributed to decreased vitamin D intake. The most likely explanation for the decreased calcitriol concentrations found in rats fed high fat diets is a reduction in calcitriol synthesis that is secondary to the increase in FGF23 (Figure 3), which is known to inhibit 1-alpha-hydroxylase and activate 24-alpha-hydroxilase in the kidney [48]. 

The low calcitriol concentrations associated with energy-dense diets may also have repercussions when considering the origin of the elevated FGF23 found after feeding high fat. As explained above, leptin stimulates FGF23 secretion by potentiating the stimulatory effect of calcitriol on FGF23, but since rats that are fed high fat diets have very low calcitriol concentrations, the mechanism whereby leptin could increase FGF23 is not clear.

Patients with CKD-MBD typically have low calcitriol levels. Low vitamin D concentrations have been associated with cardiovascular disease [49], inflammation [50], endothelial dysfunction [51], and an increased risk of bone fractures [52]. Thus a further reduction in calcitriol secondary to high fat intake would seem counterproductive in the CKD-MBD patient. 

### 2.7. Energy-Dense Diets and Vascular Calcification

In experimental models (obese Zucker rats), obesity and MS have been shown to promote uremic VC [45]. Moreover, these findings have been extended to non-obese rats that are fed high fat diets [21,53]. 

**Figure 4:**
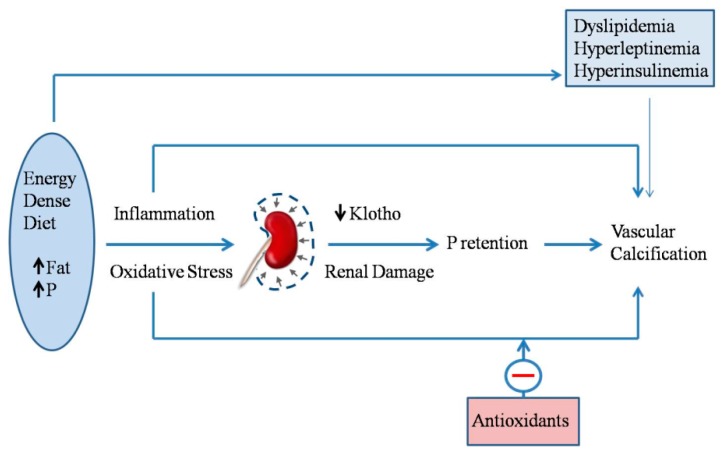
Energy-dense diets, that are rich in fat and phosphate (P), elicit inflammation and oxidative stress. In addition to the direct effects of inflammation and oxidative stress on vascular calcification, they also affect the kidney, inducing renal damage and decreasing klotho expression, thus resulting in P retention, which in turn, also promotes vascular calcification. Other biochemical changes related to eating energy-dense diets (e.g., dyslipidemia) are also likely to promote vascular calcification. Finally, antioxidants (e.g., vitamin E) have been shown to protect against vascular calcification in uremic animals eating energy-dense diets.

Phosphorus retention that is secondary to high fat intake can be a significant factor in the development of vascular and other soft-tissue calcifications (Figure 4). In patients with CKD-MBD, elevated serum P plays a major role in the development of VC [54]. Phosphate promotes VC through a series of mechanisms, including increased serum CaxP product, which leads to precipitation of Ca salts, and the phenotypic transdifferentiation of vascular smooth muscle cells (VSMCs) to osteogenic cells [11,55]. 

It is interesting to note that eating high fat diets promotes VC, even though these diets decrease circulating calcitriol concentrations. Since plasma calcitriol is very low in rats that are fed high fat, it could be speculated that treatment with calcitriol may have beneficial effects. However, the experimental data contradict this hypothesis and demonstrate that treatment with calcitriol at doses sufficient to control secondary hyperparathyroidism is clearly deleterious [53,56]. The negative actions of calcitriol are probably related to an increased P load from feeding high fat diets, which would override any beneficial effect that restoring plasma calcitriol concentrations may have on vascular health. In fact, since uremic rats fed high fat and treated with calcitriol experience such severe VC, it could be speculated that without the decrease in plasma calcitriol observed after feeding high fat diets, P retention and VC would have been enhanced. 

In addition to the modulation of P metabolism, biochemical derangements associated with metabolic syndrome may also play a role in VC that are associated with the intake of energy-dense diets (Figure 4). Hyperleptinemia is thought to increase cardiovascular risk by promoting the osteogenic differentiation of VSMCs in ApoE-deficient mice [57]. Moreover, in humans, plasma leptin levels have been found to be associated with coronary artery calcification [58]. Leptin has also been shown to promote osteoblast differentiation and the mineralization of primary cultures of VSMCs and calcifying vascular cells [59]. Although the clinical association between diabetes and VC is well-established [60], the direct relationship between insulin and VSMCs calcification is somewhat controversial [61,62]. Recent evidence indicates that insulin promotes the osteoblastic differentiation of VSMCs by increasing the receptor activator of nuclear factor kappa-Β ligand (RANKL) expression through extracellular signal-regulated kinase 1/2 (ERK1/2) activation [63]. In addition, high fat diets that are associated with hyperinsulinemic diabetes have been reported to activate an aortic osteoblast transcriptional regulatory program that is independent of intimal atheroma formation [64].

Dyslipidemia may also contribute to the severity of calcifications in uremic patients with MS [65]. It is interesting to note that histological studies clearly show that VC in uremic rats that are fed high fat is restricted to the tunica media, without evidence of the involvement of the tunica intima. Patients with advanced CKD show both intimal and medial calcifications, although arteriosclerosis seems to occur earlier in the course of the disease without being associated with lipid or cholesterol deposition [66]. 

Increased oxidative stress is a well-known complication of obesity, MS, and CKD [67,68]. Oxidant agents, including P and some uremic toxins, have been reported to promote the osteoblastic differentiation of VSMCs [69,70]. Oxidative stress has been shown to be a major factor in the development of extraskeletal calcifications, both in obese rats and in non-obese rats that are fed high fat. Moreover, the reduction in klotho that is associated with high fat intake may aggravate oxidative stress [71] and contribute to the development of VC [72].

Administration of vitamin E, one of the best known natural antioxidants, significantly reduces the degree of uremic calcification in obese Zucker rats [45] and in Wistar rats that are fed high fat diets [53]. The decrease in calcification is accompanied by a simultaneous increase in glutathione peroxydase activity, not only in plasma, but also cardiovascular tissue [45]. In summary, obesity and energy-dense diets promote extraosseous calcifications, while dietary vitamin E supplementation protects against uremic calcifications. 

Strategies to prevent or minimize VC and the progression of CKD-MBD would include P restriction and limiting the intake of energy-dense foods, because of their repercussions on P balance (e.g., P retention) and the elevation in FGF23, which accelerates progression of CKD and increases cardiovascular mortality. In addition, energy-dense diets should be restricted to minimize kidney deterioration and cardiovascular damage secondary to dyslipidemia and disturbed glucose metabolism.

## 3. Conclusions 

A major limitation when reviewing the effect of eating energy-dense diets in patients with CKD-MBD is the paucity of human clinical data. Most of the available data have been obtained in rodent models of CKD-MBD, which may not parallel the human situation. However, the few instances of human data that are available are in clear agreement with the data that is being obtained in experimental animals.

In conclusion, although energy-dense foodstuffs may have a role in preventing undernutrition and weight loss in individuals with advanced CKD; in general, these foods should be discouraged in patients with renal disease. Recent evidence indicates that besides its nephrotoxic potential, energy-dense foods promote changes in mineral metabolism (P retention, increased FGF23, decreased calcitriol, etc.) that are clearly deleterious in the context of CKD-MBD. In addition, or maybe as a consequence, these diets promote uremic extraskeletal calcification due, in part, to increased oxidative stress. 

## 4. Future Perspectives

Future investigations on this topic should aim at clinical studies to identify patients requiring dietary counselling on both P and fat intake. From a research perspective, further studies are needed to elucidate the role of energy intake on FGF23 and vitamin D metabolism, as well as to identify the mechanisms involved.
